# Automatic etiological classification of stroke thrombus digital photographs using a deep learning model

**DOI:** 10.3389/fneur.2025.1534845

**Published:** 2025-01-17

**Authors:** Álvaro Lucero-Garófano, Alicia Aliena-Valero, Isabel Vielba-Gómez, Irene Escudero-Martínez, Lluís Morales-Caba, Fernando Aparici-Robles, Diana L. Tarruella Hernández, Gerardo Fortea, José I. Tembl, Juan B. Salom, José V. Manjón

**Affiliations:** ^1^Unidad Mixta de Investigación Cerebrovascular, Instituto de Investigación Sanitaria La Fe, Valencia, Spain; ^2^Instituto de Aplicaciones de las Tecnologías de la Información y de las Comunicaciones Avanzadas (ITACA), Universitat Politècnica de València, Valencia, Spain; ^3^Unidad de Ictus, Servicio de Neurología, Hospital Universitario y Politécnico La Fe, Valencia, Spain; ^4^Servicio de Radiología, Hospital Universitario y Politécnico La Fe, Valencia, Spain; ^5^Departamento de Fisiología, Universitat de València, Valencia, Spain

**Keywords:** ischemic stroke, etiology, artificial intelligence, deep learning, segmentation, classification

## Abstract

**Background:**

Etiological classification of ischemic stroke is fundamental for secondary prevention, but frequently results in undetermined cause. We aimed to develop a Deep Learning (DL)-based model for automatic etiological classification of ischemic stroke using digital images of thrombi retrieved by mechanical thrombectomy.

**Methods:**

Patients with large vessel occlusion stroke subjected to mechanical thrombectomy between April 2016 and January 2023 at La Fe University and Polytechnic Hospital in Valencia were included. Thrombus digital images were obtained and clinical characteristics, including TOAST etiological classification as reference standard, were retrieved. Statistical analysis was performed to compare clinical characteristics between atherothrombotic and cardioembolic strokes. A DL method was designed based on two deep neural networks for: (1) image segmentation and (2) image classification including clinical characteristics. The metrics used were DICE coefficient for the segmentation network, and accuracy, precision, sensitivity, specificity and area under the curve (AUC) for the predictions of the classification network.

**Results:**

A total of 166 patients (mean age 69 [SD, 13], 67 female) were included. TOAST classification was: 31 atherothrombotic, 87 cardioembolic, and 48 cryptogenic. The segmentation network achieved an average DICE coefficient of 0.96 [SD, 0.13]. The optimal fused imaging and clinical classification network had a 0.968 accuracy [95% CI, 0.935–0.994], and AUC of 0.947 [95% CI, 0.870–1]. Cryptogenic thrombi were classified as cardioembolic (96%) or atherothrombotic (4%).

**Conclusion:**

Two convolutional neural networks perform the automatic segmentation of thrombus images and, combined with selected clinical characteristics, their accurate and precise classification into atherothrombotic or cardioembolic etiology in patients with acute ischemic stroke.

## 1 Introduction

The most commonly used classification system to differentiate ischemic stroke subtypes is the one developed by the Trial of Org 10,172 in Acute Stroke Treatment (TOAST). The different groups are: large-artery atherosclerosis (atherothrombotic), cardioembolic, small vessel occlusion, stroke of other determined etiology, and stroke of undetermined etiology (cryptogenic) (1). The correct diagnosis of ischemic stroke subtype is very important for improving clinical outcomes and preventing new events. Nonetheless, stroke classification sometimes is challenging.

One important problem faced by the neurologists is the correct classification of cryptogenic strokes, due to the unclear thrombus origin (1). Technologies such as artificial intelligence (AI), specifically deep learning (DL), could assist in classifying ischemic strokes, aiding in the development of supplementary tools for physicians (2). AI attempts to replicate human cognitive functions, while DL uses large neural networks to deal with complex regression or classification problems. One example of a deep neural network architecture is the convolutional neural network, which recognizes the visual patterns of an image and retains the main information by applying convolutions (3, 4).

AI has proven to have great applicability to aid in the diagnosis and prognosis of ischemic stroke patients. Radiomic features extracted from brain embolism regions segmented from CTA images of large vessel occlusion (LVO) stroke patients have been used as input of machine learning (ML) models for the classification of stroke subtypes. A four-center retrospective study gathered thrombus-extracted radiomic features and basic information to construct a ML model that could reliably predict cardioembolic stroke, performing better than the routine radiological method (5). In another monocentric study, DL convolutional neural networks were applied for TOAST classification (cardioembolic vs. atherothrombotic) using only radiomic features from clots in brain CTA images (6). In a different approach, a deep neural network developed to diagnose cardioembolic stroke based on chest radiographs demonstrated good classification performance and biological plausibility (7). On the other hand, ML algorithms, including a deep neural network using demographic and clinical variables, have also been created for prediction of long-term functional outcome in stroke (8). Fused imaging (MRI/CTA) and clinical DL models outperformed predictability of good reperfusion after mechanical thrombectomy (MT) (9) and functional outcome (10, 11). Although MRI and CTA medical images have been usually used to build DL models in cerebrovascular disease, a recent study used retinal photographs for screening and staging of Moyamoya disease by a DL algorithm (12).

In 1996, alteplase became the first recanalization therapy for ischemic stroke, aimed at thrombus lysis (13). However, with the advent of endovascular therapies in the last decades, such as MT (14, 15) a different approach is available to treat ischemic stroke, which also enables the study of the biological material responsible for obstructing blood flow. Since Marder’s pioneering study (16), clot composition analysis emerged as a potential diagnostic tool to gain insight into ischemic stroke etiology (17) ML algorithms have been used in the histological (18) and proteomic (19) analysis of thrombi. The largest histological study in patients from the Stroke Thromboembolism Registry of Imaging and Pathology (STRIP) found statistically significant but clinically insignificant differences between clots of cardioembolic and atherothrombotic etiologies (18).

In a study regarding the macroscopic aspect of the clots, photographs were used to visually classify the thrombi as white or red/black, with excellent inter-reader agreement for graded clot color. While white clots were significantly associated with atypical etiologies, in particular with infectious endocarditis, there was no significant difference in typical etiologies (i.e., cardioembolic vs. atherothrombotic) (20). DL can adaptively learn representative information from raw medical imaging without any preconceptions related to the human-involved feature extraction process. In the present study, we propose using DL methods to recognize complex patterns in photographs of the extracted thrombi, together with clinical characteristics of the patients, to produce accurate predictions of atherothrombotic or cardioembolic TOAST classes. For this purpose, two convolutional neural networks were used, first an image segmentation neural network, and later a binary classification neural network. To the best of our knowledge, DL models have never used photographs of retrieved thrombi to predict stroke etiology.

## 2 Materials and methods

### 2.1 Study design: patients, data retrieval, biological samples, and image acquisition

This retrospective study used a prospective registry of consecutive LVO ischemic stroke patients subjected to MT between April 2016 and January 2023 at La Fe University and Polytechnic Hospital in Valencia. The study protocol was approved by the research ethics committee (CEIm, approval #2021–577-1). Informed consent was obtained from all participating patients or their legal representatives. This study follows the Guidelines for Developing and Reporting Machine Learning Predictive Models in Biomedical Research (21).

Vascular neurologists retrieved the demographic and clinical characteristics from the medical history (age, sex, active smoking, and occurrence of diabetes, dyslipidemia, or arterial hypertension). The biological material was cerebral clots retrieved by neurointerventionalists during MT. Depending on the decision of the neurointerventionalist, based on the characteristics of the LVO, different extraction devices were used: (a) distal aspiration catheter with manual syringe, (b) balloon guide catheter plus stent-retriever, or (c) combined stent-retriever with distal aspiration catheter. The retrieved clot was either detached from the extraction device by gentle flushing with saline solution in most cases, or in some cases found in the aspiration syringe, and then preserved in saline solution at 4°C. No distinction was made between the head and tail of the thrombus, or between its shell and core. In the case of fragmented thrombi, the entire set of fragments was collected and treated as a single sample. Digital images of the thrombi were acquired once within 24 h of MT.

**Figure 1:**
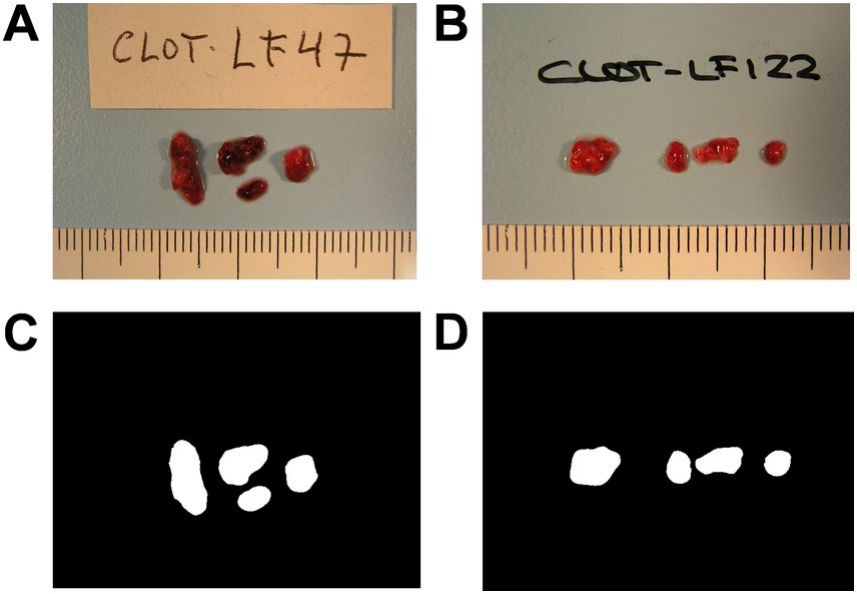
Representative images of retrieved thrombi. Images of a cardioembolic thrombus **(A)** and an atherothrombotic thrombus **(B)** as captured by the camera, and their corresponding manual segmentations, the thrombus masks **(C,D)**.

All the thrombus images were captured with the same camera (OLYMPUS^®^ CAMEDIA C-5050, Olympus Optical Co., Ltd., Tokyo, Japan), format (TIF) and resolution (640 × 480 pixels) to avoid internal variability. As the camera captured images containing thrombi and non-relevant information, a segmentation process was necessary to focus exclusively on the thrombi for image analysis. The result of this segmentation is a new binary image called mask that contains the relevant information of the original image (i.e., the thrombus; see Figure 1 for representative images of cardioembolic and atherothrombotic thrombi). The manually segmented masks were obtained using the software ITK-SNAP v3.4.0 (22). This set of images/masks was used as a ground truth to train the segmentation neural network.

Convolutional neural network was the modeling technique selected in order to combine thrombus images and patient features as input for DL categorical predictive fused models.

### 2.2 Segmentation neural network

**Figure 2:**
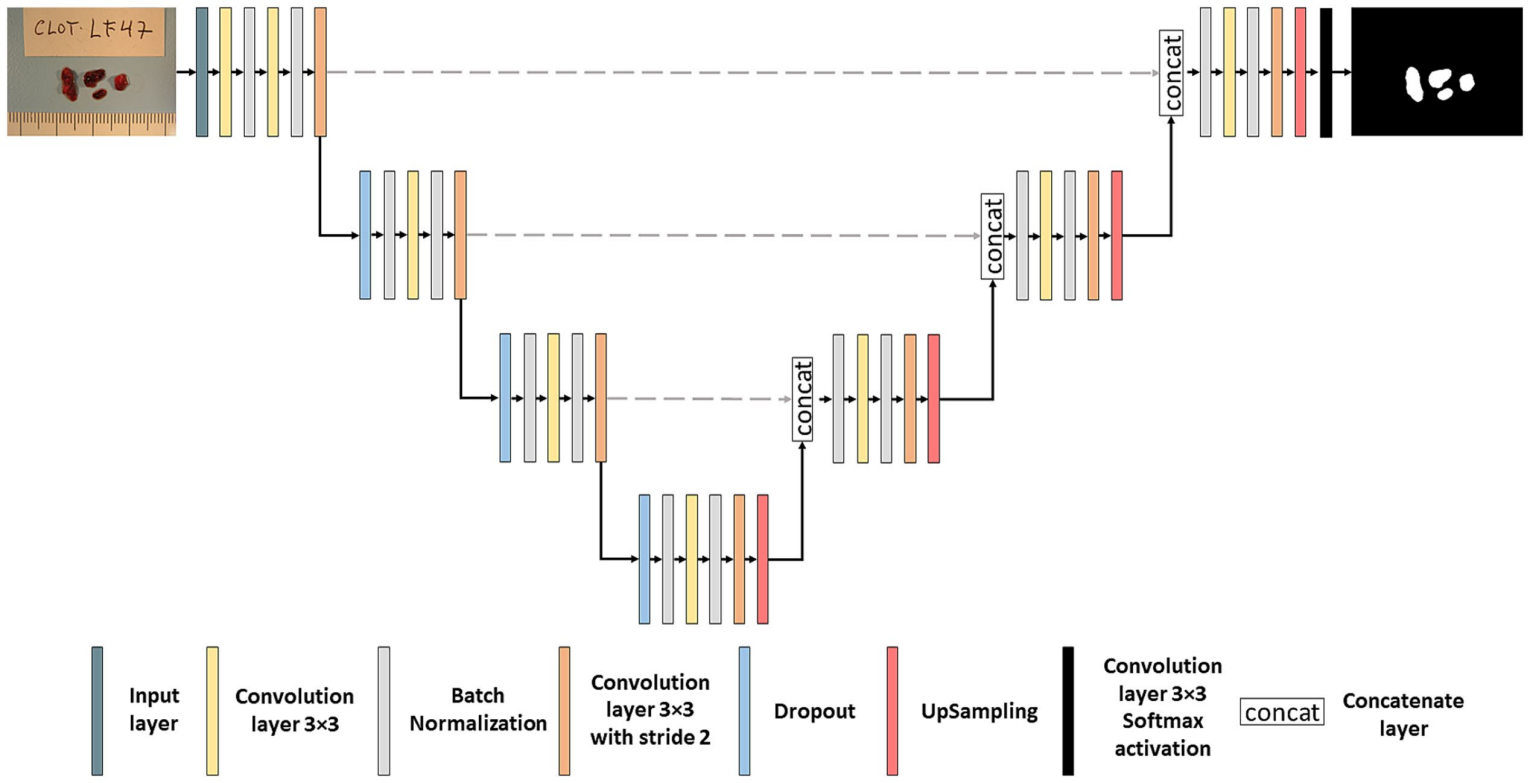
Segmentation neural network. Architecture of the U-NET neural network used for thrombus image segmentation.

Choosing the architecture of a neural network is crucial when developing a model in DL. We used one of the most widely used networks in biomedical image segmentation, the U-NET network, first developed by Ronneberger et al. (23). The U-NET consists of two parts: an encoder and a decoder which are interconnected with skip connections. The detailed architecture of the segmentation network can be seen in Figure 2. During the encoder phase, 4 blocks of convolutions layers are used, and in each block, different operations are applied such as convolution layers, batch normalization (24) and dropout (25) followed by pooling layers, effectively capturing the image’s contextual information. Batch normalization layers are used to normalize data during training, and dropout was used after pooling layers to avoid *overfitting*. In our U-NET, the kernel size utilized was 3×3 pixels, the starting number of filters was 64, and it was doubled in each down sampling step. Subsequently, the decoder comprises up-convolution layers and concatenations of the cropped feature maps extracted from the encoder part. Conversely to the encoder, the number of filters is divided in the “*up-convolutions*” of the decoder (23). All layers were activated with the ReLU function except the output layer, which was activated with the *softmax* function.

### 2.3 Classification neural network

**Figure 3:**
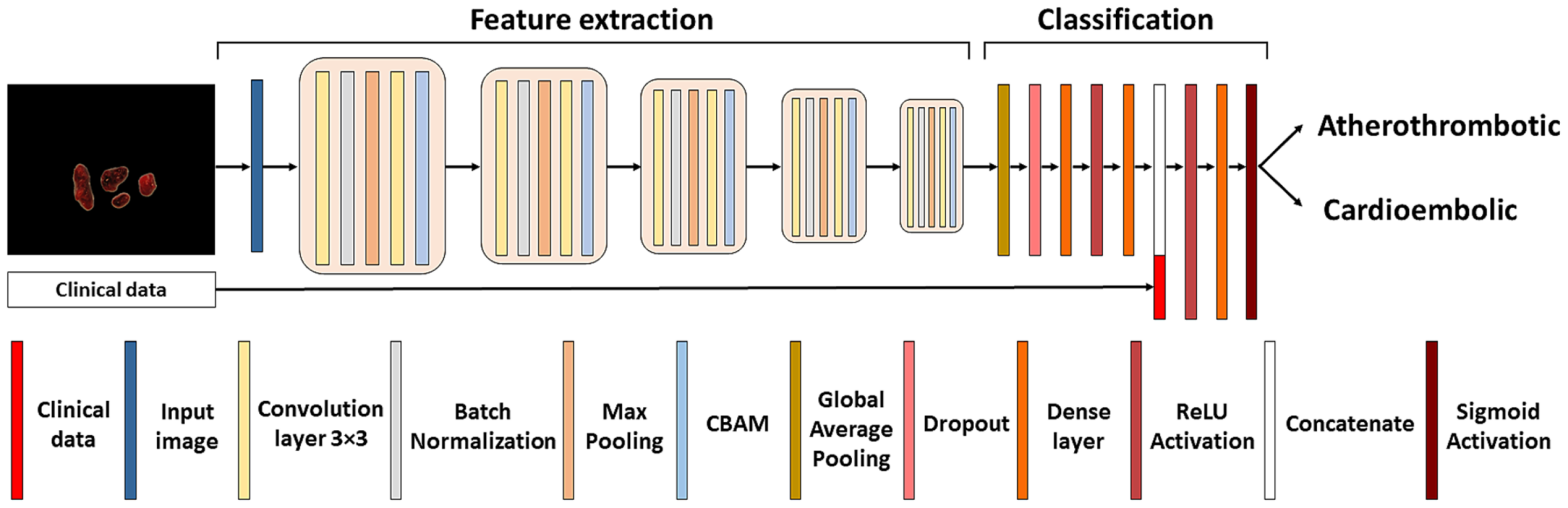
Classification neural network. Architecture of the LeNet neural network used for the etiological classification of thrombus images concatenated with patient’s demographic and clinical characteristics.

The classification network adopts an architecture reminiscent of the LeNet model (26). The input to the classification network results from multiplying the original image by the mask generated in the segmentation network. This approach selectively retains only the pertinent information for thrombus classification, discarding non-relevant details and emphasizing the macroscopic structure. The detailed architecture of the classification network can be seen in Figure 3. The network started with 64 filters, each with 3×3 pixel kernels, and this number of filters was doubled after each pooling layer, culminating in a total of 1,024 filters. Dropout and Convolutional Block Attention Module (CBAM) (27) layers were applied following each fixed resolution block to enhance accuracy. Subsequent to the feature extraction layers, a global average pooling layer consolidates all features, yielding 1,024 values per image. These values are then utilized to input a fully connected subnetwork comprising of dense layers with 128, 32 and lastly 1 neuron (all with ReLU activation, exception for the last layer which used sigmoid activation). The model’s output is a number between 0 and 1, and the image was classified as atherothrombotic or cardioembolic according to a set threshold.

Several experiments were conducted to improve the accuracy of the model. The first model exclusively used as input data the thrombus image and the mask. After the creation of this model, the concatenation of the six demographic and clinical characteristics was done. Statistical analysis was performed to find significant differences between atherothrombotic and cardioembolic etiologies regarding patient characteristics. Then, different models were created in order to find the best layer concatenation to the fully connected layer. Once the best model with the best concatenation was obtained, data augmentation techniques such as blurring or sharpening were applied to ameliorate the model’s accuracy.

### 2.4 *k*-fold cross-validation strategy

A *k*-fold cross-validation strategy was used to account for the rather small sample size. This involves dividing the dataset into *k* folds. Each fold will have a training set formed by *k*-1 folds and a test group which contains the remaining one. This process will be iterated *k* times so all data will be used as a train and test set (28). We used 5-fold cross-validation in both the segmentation and classification networks. As atherothrombotic and cardioembolic classes were unbalanced, a generator function was used to provide balanced samples to the classification network by randomly sampling the corresponding datasets.

### 2.5 Training process

The segmentation network was first trained on a subset of original images of the thrombi and their manually generated masks. Then, all the images included in the study were used. The loss function chosen was Dice loss, which employs the DICE coefficient (29). The DICE coefficient measures the overlap between two images. The optimizer utilized was Adam (30), and the number of epochs was established to 500 with 100 steps per epoch.

The classification network was trained with images whose etiology was known (i.e., atherothrombotic or cardioembolic). The loss function chosen in this network was the Binary Cross Entropy because, unlike the segmentation network, the classification operates as a binary classifier. Adamax was the optimizer employed in this network (30). The number of epochs was 1,000 with 100 steps per epoch.

DL algorithms require a substantial amount of training data, yet the disposition of annotated medical images is limited. Data augmentation was used, where some modifications are applied to the original images and improve the network’s performance (31). In our case, the modifications applied were random rotations/flips along the horizontal and vertical axes, and the application of blurring and sharpening, which affect the image edges. This process of data augmentation was applied in both segmentation and classification networks to improve their generalizability and minimize overfitting.

### 2.6 Test process

The DICE coefficient was employed to assess the performance of the segmentation network. The mask predicted by the model was generated using Test Time Data Augmentation (TTDA) (32), a technique consisting in predicting the output with different input transformations (horizontal and vertical flip in our case) to generate several predictions to later average them (after inverting the transformation). This technique is a simple way to use auto-assembling that generally improves accuracy and robustness of the network at test time.

The classification network underwent the evaluation using also the TTDA approach. The model’s input was the image multiplied by the mask generated in the segmentation network, and the output was the model’s prediction. The image was classified as either atherothrombotic or cardioembolic depending on the average numerical value returned by TTDA. A threshold was set to determine if images were classified as cardioembolic or atherothrombotic. The accuracy estimated from the training data of the model was used to determine the threshold which was stablished to 0.2. The models were evaluated three times and the results represent their average.

Once the classification predictions were obtained, which is the index test of this study, accuracy, precision, sensitivity, specificity and AUC score were used for the evaluation of the model. We used a bootstrapping approach in order to estimate the 95% confident intervals of our predictions. Bootstrap used 1,000 samples of the test set. In order to calculate these metrics, the reference standard was the TOAST classifications provided by clinicians. Performance accuracy was used to select the best model.

### 2.7 Statistical analysis

Statistical analysis was performed with the demographic and clinical characteristics obtained from the patients to observe the existence of significant differences between patients who suffered a stroke of atherothrombotic or cardioembolic etiology. The χ^2^ test was used for the categorical variables (sex, smoking, hypertension, dyslipidemia, and diabetes) and the Wilcoxon test for the non-normally distributed continuous variables (age and DICE coefficients). R software^1^ in version 4.3 was used. *p*-values <0.05 were considered statistically significant.

## 3 Results

### 3.1 Patient characteristics

**Figure 4:**
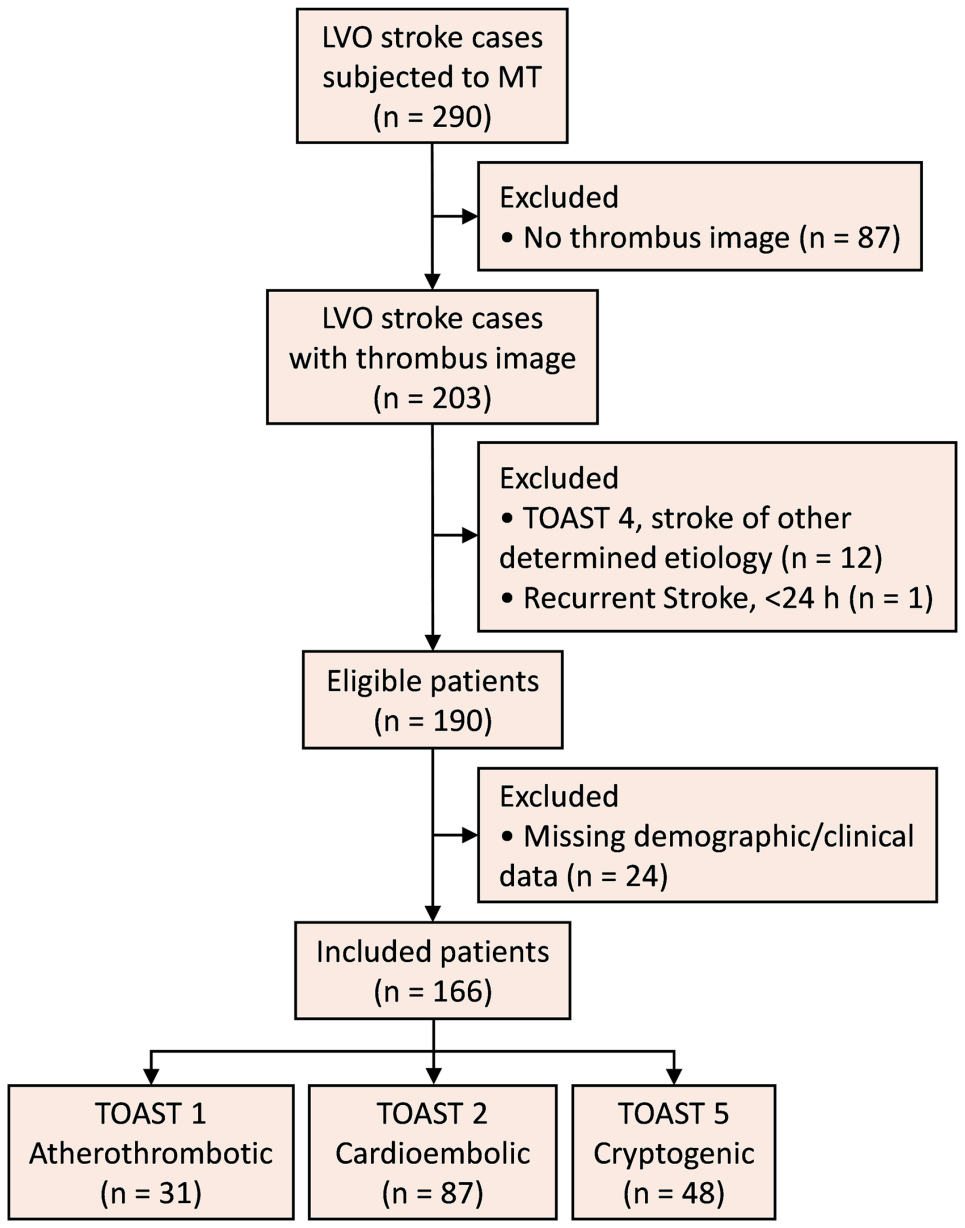
Study flowchart. LVO, large vessel occlusion; MT, mechanical thrombectomy; TOAST, Trial of Org 10,172 in Acute Stroke Treatment.

A flowchart of the study is shown in Figure 4. Out of 290 LVO stroke cases subjected to MT, 166 patients were included (age [mean ± SD] 69.11 ± 13.39 years; 67 [40.36%] female). TOAST etiologies were: 87 cardioembolic, 31 atherothrombotic, and 48 cryptogenic. Demographic and clinical characteristics are detailed in Table 1.

**Table 1:** Sample description and statistical analysis.

	Overall (*n* = 166)	Cryptogenic (*n* = 48)	Atherothrombotic (*n* = 31)	Cardioembolic (*n* = 87)	Statistic test^a^	*P*-value
Age (yr), mean ± SD	69.11 ± 13.39	65.77 ± 13.43	63.42 ± 10.76	72.98 ± 13.11	Wilcoxon	**<0.001**
Sex, n (%)					χ^2^	**0.011**
Female	67 (40.36)	24 (50.00)	5 (16.13)	38 (43.68)		
Male	99 (59.64)	24 (50.00)	26 (83.87)	49 (56.32)		
Active smoking, n (%)					χ^2^	**0.010**
No	93 (56.02)	28 (58.33)	11 (35.48)	54 (62.07)		
Yes	73 (43.98)	20 (41.67)	20 (64.52)	33 (37.93)		
Arterial hypertension, n (%)					χ^2^	0.304
No	59 (35.54)	22 (45.83)	12 (38.71)	25 (28.74)		
Yes	107 (64.46)	26 (54.17)	19 (61.29)	62 (71.26)		
Diabetes, n (%)					χ^2^	0.308
No	119 (71.69)	31 (64.58)	21 (67.74)	67 (77.01)		
Yes	47 (28.31)	17 (35.42)	10 (32.26)	20 (22.99)		
Dyslipidemia, n (%)					χ^2^	0.418
No	75 (45.18)	22 (45.83)	12 (38.71)	41 (47.13)		
Yes	91 (54.82)	26 (54.17)	19 (61.29)	46 (52.87)		

a Statistical tests were performed between atherothrombotic and cardioembolic data. SD, standard deviation; χ^2^, chi-squared test. Bold *p*-values <0.05 were considered statistically significant.

### 3.2 Image segmentation performance

In the segmentation network, the images of all 166 patients were used, regardless of stroke etiology. The proposed segmentation network had a total of 1,963,202 parameters (1,959,938 trainable) and obtained a DICE coefficient of 0.95551 ± 0.12996 without TTDA and 0.95553 ± 0.13037 using it. As can be noticed, TTDA improved results (although not statistically significant, *p* = 0.744, W = 0.310, Wilcoxon test). The average processing time of the segmentation network was 0.5 s per image.

### 3.3 Etiology classification performance

In the classification network, the images of 118 patients with known etiology were used (31 atherothrombotic and 87 cardioembolic). Three consecutive models were developed to optimize accuracy, precision, sensitivity, specificity, and area under the curve (AUC). Cardioembolic etiology was considered the positive class. Table 2 summarizes input data, data augmentation, and performance metrics (mean [95% CI]) for each model.

**Table 2:** Summary of input data, data augmentation, and performance metrics of the classification models.

	Model 1	Model 2	Model 3	Model 3^*a^
Input data	Image	Image, 6 features	Image, 3 features	Image, 3 features
Data augmentation	Rotations	Rotations, sharpening, and blurring	Rotations, sharpening, and blurring	Rotations, sharpening, and blurring
TTDA	Rotations	Rotations	Rotations	Rotations and blurring
Accuracy	0.944 (0.901–0.977)	0.958 (0.918–0.992)	0.960 (0.920–0.991)	0.968 (0.935–0.994)
Sensitivity	0.969 (0.929–1)	1 (1–1)	0.988 (0.963–1)	1 (1–1)
Specificity	0.871 (0.745–0.970)	0.839 (0.697–0.960)	0.881 (0.753–0.980)	0.881 (0.753–0.980)
AUC	0.943 (0.873–0.994)	0.960 (0.901–0.999)	0.993 (0.981–1)	0.947 (0.870–1)
Precision				
Atherothrombotic	0.910 (0.795–1)	1 (1–1)	0.964 (0.889–1)	1 (1–1)
Cardioembolic	0.955 (0.908–0.989)	0.946 (0.838–0.989)	0.959 (0.912–0.993)	0.959 (0.914–0.993)

Classification metrics are expressed as mean (95% CI). Cardioembolic etiology was considered the positive class. ^a^Model 3* is Model 3 with different TTDA configuration. TTDA, test time data augmentation; CI, confidence interval; AUC, area under the curve.

Model 1 exclusively used the original image multiplied by the mask as input. The accuracy of the model was 0.944, and the precisions were 0.910 and 0.955 for atherothrombotic and cardioembolic etiologies, respectively.

Given the possibility of accessing demographic and clinical data associated with the thrombus image, Model 2 concatenated all the six characteristics (age, sex, active smoking, diabetes, dyslipidemia, and arterial hypertension occurrence) in the dense layer with 128 neurons to the fully connected layer of the classification model. In addition, image sharpening and blurring operations were applied in the data augmentation process. The accuracy of the model increased to 0.958, and the precisions were 1 and 0.946 for atherothrombotic and cardioembolic etiologies, respectively.

Given that the addition of demographic and clinical characteristics improved the accuracy of Model 2, we decided to perform a statistical analysis of these characteristics to find out statistically significant differences between patients who suffered from a stroke of cardioembolic or atherothrombotic etiology (Table 1, characteristics with a *p*-value <0.05 have been highlighted). In the light of the statistical analysis results, only the 3 significantly different characteristics (age, sex, and active smoking) were included in Model 3, and they were concatenated in the same layer that Model 2. The model increased its accuracy from 0.958 to 0.960. The precisions were 0.964 and 0.959 for atherothrombotic and cardioembolic etiologies, respectively.

Finally, we tested Model 3 with a different TTDA configuration consisting of blurring the images to see its effect not only in the training process, but also in the test. The accuracy of this optimal Model 3^*^ increased to 0.968, and the precisions for atherothrombotic and cardioembolic etiologies were 1 and 0.959, respectively. AUC for cardioembolic (positive) prediction was 0.947. As can be noticed, the blurring at TTDA improved the accuracy metrics, thus suggesting that color information was more relevant for classification that texture information. The concatenation of the 3 patient characteristics in Model 3^*^ was performed in the dense layer with 32 neurons. The parameters for this model were 4,874,922 (4,872,938 of them were trainable).

### 3.4 Classification of cryptogenic thrombi

**Figure 5:**
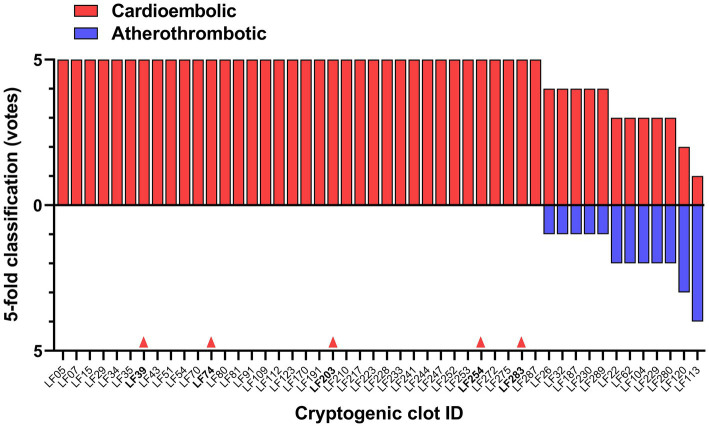
DL-model classification of cryptogenic thrombi into cardioembolic or atherothrombotic. The votes of the 5-fold model for each clot are shown. Red arrows indicate 5 cases clinically confirmed as cardioembolic stroke by further diagnostic workup.

Using Model 3^*^, the best performing classification network for atherothrombotic and cardioembolic thrombi, we conducted a preliminary experiment to evaluate the 48 images of thrombi with cryptogenic etiology (together with age, sex, and active smoking data) and predict their classification. Cryptogenic thrombi were evaluated by the 5 folds of the model and the final result was the vote of the majority of the folds. The network classified 46 images as cardioembolic and 2 images as atherothrombotic (Figure 5). This suggests that cryptogenic thrombi are more similar to cardioembolic thrombi (95.83%) than atherothrombotic thrombi (4.17%), which is in good agreement with the higher incidence of cardioembolic etiology. In 36 out of 46 cases (78.26%) the prediction of cardioembolic origin was unanimous. After further diagnostic workup, 5 out of the 36 unanimous predictions were clinically confirmed as cardioembolic etiology (Figure 5).

## 4 Discussion

In this study, we have developed and optimized a method to automatically segment stroke thrombus images from digital photographs and to accurately classify them, together with clinical characteristics, into atherothrombotic and cardioembolic categories. Our results show the usefulness of using DL on thrombus photographs for typical etiology classification, when compared to visual classification by expert interventional neuroradiologists (20). High accuracy of our best model (0.968) outperformed accuracies reported in previous studies using AI to predict cardioembolic or atherothrombotic origins based on different clot features. Combination of DL convolutional neural network and radiomics of brain embolism regions segmented from the CTA images predicted stroke subtype with 0.8929 accuracy (6). A ML model using thrombus-extracted radiomic features from CTA images and basic information predicted cardioembolic stroke with 0.904 accuracy (5). With regard to retrieved thrombi, ML models classified cardioembolic versus atherothrombotic with 0.77 accuracy on the basis of clot histomics (33), 0.883 accuracy based on clot proteomics (19), and 0.889 accuracy when metabolomics features were used as etiology predictors (34). On the other hand, classification of cardioembolic stroke based on a DL neural network using chest radiographs showed 0.844 accuracy (7).

The segmentation network with the U-NET architecture has demonstrated high performance in various medical imaging tasks, such as segmentation of brain tumors or skin lesions (35, 36). The average DICE coefficient obtained in our case was 0.955, proving its good performance. This allowed reliable automatic segmentation of the thrombus from the whole photograph, in contrast to time-consuming and less accurate manual segmentation carried out to delineate the cerebral embolism region in the CTA image on the patient’s head before extracting radiomic features for etiology prediction (6).

Regarding the classification neural network, our best model achieved not only a high accuracy of 0.968, but also high precisions of 1 for atherothrombotic and 0.959 for cardioembolic cases. These results suggest that the proposed method can effectively classify thrombi as either atherothrombotic or cardioembolic. The architecture of the model was inspired in the well-known architecture proposed by LeCun et al. (26). The main difference in the present study was the application of dropout, Adamax optimizer, convolutional block attention module (CBAM) (27), and the change of the activation function in the last layer from *softmax* to *sigmoid* due to our approach, which is a binary classification.

Classification metrics were improved in the present study by combining imaging and selected clinical data in the DL model. This kind of fused DL models have not been previously used for stroke etiology prediction, but outperformed separate imaging or clinical models (and traditional risk-scoring by expert neurologists) when used in the prediction of functional ischemic stroke outcome (10, 11, 37) and good reperfusion after endovascular treatment (9). Performance of more complex neuronal networks combining clot macroscopic imaging, omics features and patient clinical characteristics deserves further research.

Different data augmentation options were used. We included rotations as the orientation of the clots was somewhat random at the moment of image acquisition. Blurriness is also a realistic artifact as sometimes the camera can be not totally focused. Of note, our best performing model applied blurring after the process of data augmentation. The improved performance by application of blurring may indicate that the network is using mainly color information from the image rather than texture information. Previous studies have compared the proportions of fibrin and cellular components (i.e., chromatic aspect) in atherothrombotic and cardioembolic thrombi, but there is no consensus. Some studies report that cardioembolic thrombi contain more red blood cells (red thrombus) than atherothrombotic thrombi (38, 39), while others conclude the opposite (40, 41). Of note, a recent meta-analysis including 21 studies found that fibrin composition is significantly higher (white thrombus) in strokes of cardioembolic and cryptogenic origin than in strokes of non-cardioembolic origin (42).

Cryptogenic thrombus and their clinical characteristics were introduced as input data into the classification network as a proof-of-concept for the classification of thrombi with unknown origin. The findings of this experiment indicate that the classification network tends to unanimously categorize in most cases cryptogenic thrombi as cardioembolic rather than atherothrombotic. A previous study also more likely predicted a cardioembolic origin in cryptogenic thrombi by applying a histomics-based ML model (33). These results are in line with similarities in thrombus histology, interventional and clinical outcome parameters previously observed between cryptogenic and cardioembolic thrombi, when compared to non-cardioembolic thrombi (43), thus supporting the hypothesis that the majority of cryptogenic strokes are actually cardioembolic. However, this should be taken with caution, as bioinformatic analysis of clot transcriptomes from different TOAST etiologies showed that gene expression of cryptogenic thrombi was not clustered toward only one group, but showed expression patterns related to both atherothrombotic and cardioembolic etiologies (44). The use of DL offers a new research avenue for uncovering cryptogenic stroke. The next step in this process should be to ensure that cryptogenic strokes, which were initially classified as a cardioembolic stroke by the model, are clinically re-evaluated to detect any underlying cause of cardioembolic stroke and thus confirm the diagnosis. Interestingly, at present 5 out of the 36 cryptogenic thrombi unanimously predicted as cardioembolic by the model have been clinically confirmed after further diagnostic workup. This supports the need to maintain the diagnostic workup effort in the remaining 31 patients.

Regarding atherothrombotic strokes, there has been in recent years an increasing interest in the application of AI in carotid plaque detection using ultrasound, CT scans, and MRI. Deep learning models based in CNNs have been demonstrated to be a valid help in identifying the characteristics of vulnerable and potentially symptomatic plaques, increasing the accuracy of imaging detection, or simply speeding up the diagnostic process, in order to prevent future cerebral ischemic events (45).

Although this study provides a DL classification model that shows translational promise in stroke diagnosis, it has limitations. Regarding the handling of thrombotic material, different MT devices and techniques were used to obtain clot samples with different degrees of fragmentation, which were stored in cold saline solution for different periods of time until image acquisition within 24 h, potentially affecting the morphological characteristics of the thrombi. It is a monocentric study with a limited sample size, although higher than in other studies using ML and clot histomics (33), proteomics (19), or radiomics (6). Some patients who underwent MT during the recruitment period were excluded because thrombus image or demographic/clinical data were not available, which may have introduced selection bias. However, the proportions of cardioembolic, atherothrombotic and cryptogenic etiologies in the patients finally included were quite similar to those expected in the TOAST stroke subtype classification in clinical practice (46). Data augmentation (image modification) techniques and a *k*-fold cross-validation strategy were used to account for the limited number of images available and to minimize overfitting. These methods proved useful in improving the performance of algorithms for classifying stroke subtypes (33) or predicting stroke functional outcome (37). Another limitation is the representativeness of the sample. The limited sample size has the potential to complicate the generalizability of the model, even with the use of techniques such as data augmentation and k-fold cross validation, given their dependence on the original dataset. To overcome this problem, a highly standardized protocol was developed to augment the dataset not only in our center, but also in different centers. Therefore, the present results should be extended in a multicentric study to increase the sample size and perform external validation of the model. For this purpose, it would be important to standardize data acquisition in all centers of a collaborative network. Finally, this DL model can only be used for patients from which a clot is retrieved. However, the estimated population eligible for endovascular treatment is ∼10% of stroke admissions (47).

## 5 Conclusion

In conclusion, two convolutional neural networks have been built for the automatic segmentation and highly accurate and precise etiological classification of thrombus images from patients with LVO acute ischemic stroke. This innovative approach should be validated in a multicentric study with a larger sample size. It has translational potential to serve as a complementary diagnostic support tool for vascular neurologists, thereby improving patient classification and enhancing decision making, particularly for secondary prevention of new events.

## Data Availability

The raw data supporting the conclusions of this article will be made available by the authors, without undue reservation.

## Footnotes

^1^ http://www.r-project.org
